# Salivary pH changes under the effect of stainless steel versus elastomeric ligatures in fixed orthodontic patients: a single-center, randomized controlled clinical trial

**DOI:** 10.1186/s12903-021-01906-4

**Published:** 2021-10-22

**Authors:** Hend Abulkarem Abdullah Al-Haifi, Ramy Abdulrahman Ali Ishaq, Maged Sultan Abdullah Al-Hammadi

**Affiliations:** 1 Present Address: Department of Biological and Preventive Sciences, Faculty of Dentistry, University of Science and Technology, Sana’a, Republic of Yemen; 2 Department of Orthodontics, Pedodontics, and Preventive Dentistry, Faculty of Dentistry, Sana’a University, Sana’a, Republic of Yemen; 3 Department of Preventive Dental Sciences, Faculty of Dentistry, Jazan University, Jazan, Saudi Arabia

**Keywords:** Enamel demineralization, Elastomeric ligature, Stainless steel ligature, Salivary pH

## Abstract

**Background:**

Fluctuations in pH of saliva during a prolonged treatment course influences the enamel demineralization progress, which is one of the complications of fixed orthodontic treatment. This randomized clinical trial aimed to evaluate and compare the short-term effects of stainless steel (SS) versus elastomeric (EM) ligatures on salivary pH in patients scheduled for fixed orthodontic treatment.

**Methods:**

Seventy participants were enrolled in the study (54 female, 16 male) aged 19–36 years who met specific inclusion criteria. They were randomly selected and allocated into two equal groups through computer-generated randomization. All patients received fixed orthodontic treatment using conventional orthodontic brackets. Two commonly used archwire ligature methods were used: SS and EMs. An unstimulated (resting) salivary sample was collected before tying of the ligatures at T0 (baseline), 2 weeks, 6 (weeks), and 12 (weeks). Salivary pH was measured using a digital pH meter. The level of significance was set at p value < 0.05.

**Results:**

The salivary pH level was stable between T0 and T1 (6.72 ± 0.14), then significantly and progressively increased from T1 to T2 (6.78 ± 0.13) and from T2 to T3 (6.81 ± 0.14) with (p < 0.05) in the SS group. In the EM group, the salivary pH level was significantly decreased in all follow-up periods; T0 (6.77 ± 0.16), T1 (6.72 ± 0.14), T2 (6.67 ± 0.13) and T3 (6.64 ± 0.13).

**Conclusion:**

The EM ligatures showed a significant decrease in salivary pH to an unfavorable level, which increased the risk of enamel demineralization. Therefore, EMs as ligature material is preferably should not be recommended in patients with high caries index or inadequate oral hygiene.

*Trial registration* ANZCTR.org. (ACTRN12618001647224) http://www.anzctr.org.au/ACTRN12618001647224.aspx. Registration Date: 5/10/2018, “Retrospectively registered”.

## Background

Malocclusion is considered as the third most common oral problem following dental caries and periodontal diseases [1, 2]. Most malocclusions are treated by fixed orthodontic appliances as a second phase following orthopedic treatment, as a comprehensive treatment after cessation of growth, or as a preparatory stage for orthognathic surgery [3]. Treatment of malocclusion with fixed orthodontic appliances is estimated to last from 18 to 36 months [4, 5]. As they are termed “fixed appliances,” they are fitted permanently to the teeth, creating plaque-retentive areas around the bracket wings. This increases chances for plaque accumulation and bacterial colonization. The main formed colonies of specific interest are the acid-producing bacteria *Streptococcus mutans* and *Lactobacilli* [6–11].

Plaque harboring around the brackets is also influenced by the archwire ligation material [7, 10, 12–14]. Stainless steel (SS) and elastomeric modules (EM) are the most commonly used materials to secure archwire to the bracket slots [14]. They have different properties, including their surface topography.

SS ligatures demonstrated less plaque retention compared to EM and are, therefore*,* better for oral hygiene maintenance [14]. Higher levels of acidogenic bacteria are detected with EM ligatures*,* most noticeably *S.mutans* and *Lactobacilli* [10, 15]. This contributes to the drop in salivary pH levels during orthodontic treatment [16, 17]. The choice to use either ligation method has been shown to be a matter of personal preference of the practitioner and is influenced by the patient choice.

Salivary pH is one indicator of caries susceptibility of the individual patient. The risk of enamel demineralization is increased if salivary pH drops below the critical value (pH = 5.5) [18–20]. This drop contributes to the formation of white spot lesions reported to occur in around 50% of the orthodontic patients [21–24].

The effect of fixed orthodontic appliances on salivary pH has been investigated in previous studies without reporting which type of archwire ligation material was used. In an observational study, Zogakis et al. [25] demonstrated a significant decrease in pH of saliva six weeks after fitting the appliance. Different results were presented by Peros et al. [26] who reported an increase in salivary pH at 6, 12 and 18 weeks after fixed orthodontic therapy. Bonetti et al. [16] reported that no changes in salivary pH values occurred under the effect of fixed orthodontic treatment. To the best of our knowledge and according to the available literature, no randomized clinical trial (RCT) has evaluated the effect of SS and EM ligation materials on pH of saliva [27].

### Specific objectives

The aim of this trial was to evaluate and compare the short-term effects of SS and EM ligatures on salivary pH in patients treated with fixed orthodontic appliances.

## Methods

### Trial design

This investigation was a two-arm parallel-group randomized controlled clinical trial. No changes were introduced to the trial following commencement.

### Participants, eligibility criteria, and settings

The selection criteria included subjects requesting orthodontic treatment, aged between 19-36 years, had good oral hygiene, and were periodontally healthy (plaque index ≤ 1). Patients with habits (mouth breathing, smoking, or any chewing habits), chronic or systemic diseases or chronic medication intake were excluded. The deterioration of oral hygiene level after recruitment was considered an exclusion criterion.

Patients were recruited from the Department of Biological and Preventive Sciences, College of Dentistry, University of Science and Technology, Sana’a, Republic of Yemen. Consecutive patients were examined by the primary researcher. Those meeting the selection criteria were invited to participate. Informed consent was signed after the nature of the study was explained.

### Intervention

This study follows the guidelines of the Consolidated Standards of Reporting Trials statement [28]. Ethical approval was obtained from the ethics committee of the University of Science and Technology (Registry No: EAC/UST126). All participants received a standard protocol of oral hygiene instructions and motivation (according to Bass technique) using tooth paste containing fluoride.

All subjects were treated with straight wire appliances using MBT bracket system (SIA, Italy). Alignment and leveling were initiated with round nickel-titanium archwires and treatment proceeded as required for each patient.

The sample consisted of two groups, each included 35 subjects. They received archwire ligation (SIA, Italy) with either SS (Group A) or EM (Group B) based on the randomization technique followed. The ligations were replaced every four weeks during follow-up visits.

### Saliva collection

Unstimulated (resting) whole salivary samples were collected according to the protocol derived from the World Health Organization/International Agency of Research [29] as follow.

The samples were obtained in the morning between 9 A.M. and 12 P.M. using the passive drooling method. The subject was seated in the dental chair and instructed to allow saliva to pool in the mouth passively for five minutes, then drool it into a graduated plastic sterile tube. These samples were instantly transferred into a reservoir container and immediately sent to the laboratory for salivary pH measurement. The laboratory technician received the salivary samples that were coded and labeled without any indications of intervention details.

### Outcomes

The outcome of this study was to measure the pH of the salivary samples collected from the patients at four time points. The baseline value was measured before the placement of ligature materials (T0). T1, T2 and T3 were measured at 2, 6 and 12 weeks from T0. The purpose was to evaluate and compare the change in salivary pH values with the introduction of two different types of ligatures in two randomly assigned groups.

Measurements were performed in the Laboratory of Drugs and Medicine, College of Pharmacy, University of Science and Technology, Sana’a, Republic of Yemen. A digital handheld pH meter with incorporated automatic temperature compensation (3510, JENWAY, UK) was used. The procedure was conducted according to the manufacturer’s instructions and included the following steps: (a) Calibration was performed by freshly prepared standard buffer solutions at pH = 7 and 10. (b) For pH measurement, the probe sensor was fully immersed inside the sample for 30s to get a stable final reading. (c) Disinfection was achieved by washing the meter under running water to remove any remnants. It was then cleaned with alcohol and allowed to dry [30].

### Sample size calculation

The required sample size was calculated using the G*power software. Based on previous studies with a mean difference of 0.1 unit change in pH and a ± 0.14 standard deviation (SD) with a power of 80% and α = 0.05, a minimum number of 35 participants was required for each group [16, 31].

### Randomization (random number generation, allocation concealment, implementation)

A total of 70 participants (54 female and 16 male) ranging in age from 19 to 36 years were randomly selected with a 1:1 allocation ratio. Randomization was accomplished with random permuted blocks of 70 participants with the allocations concealed in sequentially numbered, opaque, sealed envelopes. The clinic assistant was responsible for generating the allocation sequences, preparing the enclosed envelopes in sequence numbers, enrolling the participants, and assigning them into their groups.

### Blinding

Blinding of the patients and investigator to the intervention was not implemented. Instead, this applied to the laboratory technician and the statistician.

### Statistical analysis

The data were statistically analyzed using statistical software (SPSS version 25; Armonk, NY: IBM Corp). Data included age, gender, type of archwire ligature (SS or EM), and salivary pH at T0, T1, T2 and T3. The Shapiro–Wilk test was applied to verify the normality of distribution of the examined variable. The t-test for independent samples was applied for the comparison between groups of archwire ligation. Intra-group difference comparisons between T0 and T1, T2 and T3 were carried out with the t-test for paired samples. Repeated measures ANOVA was used to make comparisons between T0, T1, T2 and T3, and the level of significance was set at p < 0.05.

## Results

### Baseline data

Recruitment began in January, 2018 and ended in March, 2019. Baseline demographic and clinical characteristics for each group are shown in (Table 1.) The data relative to salivary pH were normally distributed.

**Table 1:** Baseline demographic characteristics data for participants in both groups

Study characteristics	Stainless Steel	Elastomeric	Total
Number of participants (who received the allocation intervention)	31	31	62
Age (y)—mean (SD)	20.45 ± 4.26	20.48 ± 3.92	20.47 ± 4.60
Gender distribution (Male / Female)	6/25	6/25	12/50

*y* years, *SD* standard deviation

### Participant flow

**Fig. 1:**
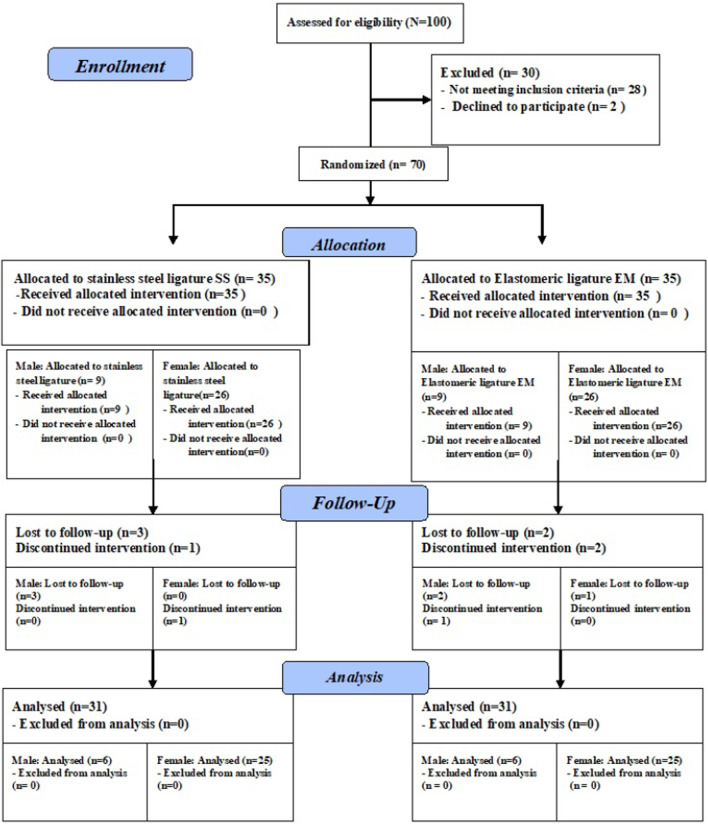
The CONSORT flow chart diagram for the follow up of participants throughout the trial

The flow of participants throughout the trial is demonstrated in the flowchart (Fig. 1). Eight participants did not receive the allocated intervention for various reasons, including a lack of follow-up and discontinuation of treatment.

### Outcomes and estimation

Significant changes in salivary pH values were observed (p < 0.05) for both ligation materials compared to the baseline values (T0). The EM group showed a significant decrease of salivary pH value in all assessment time points. Regarding the SS group, a significant increase of salivary pH value was observed at 6 (T2) and 12 (T3) weeks (Table 2). Repeated ANOVA measured for multiple comparisons revealed a significant difference in salivary pH value between T0, T2 and T3 (Table 3).

**Table 2:** Longitudinal changes in salivary pH values for each group from T0 to T3

pH value at	T0		T1		T2		T3		p value		
Ligature	Mean	SD	Mean	SD	Mean	SD	Mean	SD	T0-T1	T0-T2	T0-T3
Stainless Steel	6.72	0.14	6.72	0.14	6.78	0.13	6.81	0.14	0.81NS	0.00*	0.00*
Elastomeric	6.77	0.16	6.72	0.13	6.67	0.13	6.64	0.13	0.00*	0.00*	0.00*

*T0* baseline, *T1* 2 weeks, T2 6 weeks and T3, 12 weeks from T0, *NS* indicates nonsignificant

*Significant p value < 0.05

**Table 3:** Multiple comparison for salivary pH mean difference from T0 to T3

Time	T1	T2	T3	p value		
Comparison	T0–T1	T0–T2	T0–T3	T0–T1	T0–T2	T0–T3
Mean difference	0.03	0.02	0.02	0.08NS	0.00*	0.00*
P value	0.70	0.02	0.04	0.00*	0.00*	0.00*

*T0* baseline, *T1* 2 weeks, *T2* 6 weeks and T3, 12 weeks from T0, *NS* indicates nonsignificant

*Significant p value < 0.05

### Harm

No serious harm was inflicted upon the participants other than moderate marginal gingivitis associated with fixed orthodontic treatment.

## Discussion

### Limitations

Blinding of the investigator and participants was not feasible. Short-term follow-up for 3 months during alignment and leveling stage was conducted, since only brackets, archwires and ligatures used excluding other method, such as, elastics, power chain and coils, among others. Bacterial colonization was not measured and it is recommended to be done in any future studies to support the significant drop in salivary pH level. Type of drinks, food, oral hygiene and other confounders are to be considered in future studies with larger sample size.

### Generalizability

The above findings may be applicable to other populations. The average salivary pH values ranged from 6.5 to 7.5, with no difference between communities and populations [30, 32].

### Interpretation

The importance of preservation of enamel integrity during orthodontic treatment urges orthodontists and researchers to improve the appliances used to decrease the chances for plaque accumulation and subsequent enamel demineralization.

In their systematic review, Freitas et al. [33] reported that fixed orthodontic appliances affect the quality and quantity of oral microbiota with a significant increase of acid producing bacteria, particularly *S. mutans* and *Lactobacilli*, which contributes to the drop in salivary pH level during the course of treatment.

No previous study has evaluated the effect of archwire ligation materials on salivary pH. Comparison of the current study’s findings with those of previous studies was not possible. Several previous studies evaluated the effect of fixed orthodontic appliance on salivary pH. They compared the changes before and after treatment without specifying which type of ligature materials were used [12, 16, 17, 25, 31, 33–40].

The effect of archwire ligation materials (SS and EM) have been previously evaluated on oral biomarkers other than salivary pH, including plaque, gingival, bleeding indices, and microbial colonization with a split mouth design [10, 13–15]. Even in the last three years several studies were conducted and none of them specify the type of ligature used. Alshahrani et al. [41] reported significant reductions in the salivary flow rate and pH two months after commencing fixed orthodontic treatment and AlHudaithi and Alshammery [42] found significant reduction in salivary pH after four to five weeks of de-bonding or at the retention period. At the same time, Anu et al. [43], Dallel et al. [44], and Kouvelis et al. [45] concluded that salivary pH did not significantly change between the studied time points during fixed orthodontic treatment.

The EM group demonstrated a significant decrease in salivary pH level in all stages. By comparison, the SS group showed an increase after T1. The current study findings agree with the results of Forsberg et al. [14] that demonstrated a higher level of *S. mutans* and *Lactobacillus* on EM ligature compared to SS for 12 orthodontic patients. They recommended that EM should be avoided for patients with inadequate oral hygiene.

Türkkahraman et al. [10] evaluated the microbial colonization between two archwire ligatures with split-mouth study at the early stages of orthodontic treatment for 21 orthodontic patients at three different times. They reported that EM ligature had slightly more microbial colonization of *S. mutans* and *Lactobacilli* compared to SS. However, the difference was not significant.

Alves de Souza et al. [13] conducted a study to evaluate microbial colonization using polymerase chain reaction analysis of two archwire ligations, EM and SS before fixed orthodontic appliance and after 6 months for 14 patients with split mouth design. They reported that EM ligatures were associated with higher scores of microbial plaque index than SS ligatures.

In comparison, Sukontapatipark et al. [8] conducted an experimental study by scanning electron microscopy, evaluated microbial colonization on two types of archwire ligation, EM and SS, for 20 patients at three time intervals over a period of three weeks. They demonstrated that the ligation material did not influence the microbial morphotypes.

EMs are considered an organic material in their composition which would be more favorite for bacterial colonization than SS, which is an inorganic material with an inert metal surface [46, 47]. The differences in surface tomography and structural characteristics of elastomeric and steel ligature wires may also be a factor that enhances the bacterial colonization on the organic and porous surfaces of EM. These are diminished with SS, which has inorganic and plant surfaces [48, 49]. The discrepancies may be attributed to the difference in study design and duration, sample size, methods of saliva collection, and tools for measuring salivary pH.

Females’ participants most often apply for orthodontic than males [50], more often maintain attendance, and commitment to orthodontic clinics and treatment instructions. Unstimulated (resting) saliva was specified for collection. This is an oral secretion in contact with teeth and is a long-lasting rather than stimulated saliva.

The clinical significant of study results demonstrated via a short-term evaluation that EM is a cause of a drop in salivary pH, which constitutes a risk indicator for enamel demineralization and the formation of white spot lesions throughout treatment with fixed orthodontic appliances.

Preventive approaches relating to changes in the oral environment in orthodontic patients were established. Checking the pH of saliva can be valuable as part of an overall clinical assessment especially for patients with high caries index.

## Conclusion

Early in orthodontic treatment, EM had a significant effect on salivary pH compared with SS ligature, lowering it to unfavorable levels. Therefore, the EM as ligature material should preferably not to be recommended in orthodontic patients with high caries index or inadequate oral hygiene.

## Data Availability

Any of the supporting data in this manuscript are available in any time.

## Abbreviations

EM: Elastomeric Modules
SS: Stainless Steel
RCT: Randomized Clinical Trial
